# Postoperative pancreatic fistulas after pancreaticoduodenectomy for malignancy: A Northeast Indian tertiary cancer center study

**DOI:** 10.1002/jgh3.12609

**Published:** 2021-08-03

**Authors:** Joydeep Purkayastha, Srinivas Bannoth, Abhijit Talukdar, Bibhuti Bhusan Borthakur, Deepjyoti Kalita, Gaurav Das, Kiran Kamalasanan

**Affiliations:** ^1^ Department of surgical oncology Dr. B. Borooah Cancer Institute Guwahati India

**Keywords:** malignancy, pancreas, pancreatic fistula, pancreaticoduodenectomy

## Abstract

**Background and Aim:**

Postoperative pancreatic fistula (POPF) is an important cause of major morbidity and mortality after pancreaticoduodenectomy. We intend to estimate the incidence and study the risk factors and outcomes of patients who developed this dreaded complication.

**Methods:**

This is a retrospective observational study. We included all patients who underwent pancreaticoduodenectomy at a specialized surgical unit of a single tertiary care cancer center in Northeast India. The period of study was from 23 April 2012 to 27 December 2019. The 2016 update on the definition of POPF by the International Study Group for Pancreatic Fistula was used to define the complication. Chi‐square test and Fischer's exact test were applied to categorical variables. *t*‐test was used to quantify mean difference among continuous variables. *P* value <0.05 was considered statistically significant at 95% confidence interval.

**Results:**

A total of 59 patients underwent pancreaticoduodenectomy during the study period with almost equal distribution among males and females (29 and 30 patients respectively). The mean age of the patients was 54.0 years (range 20–72). Grade A, B, and C pancreatic fistulas were seen in five (8.5%), three (5.1%), and two (3.4%) patients, respectively. Preoperative hyperbilirubinemia, pancreatic duct size ≤3 mm, hypoalbuminemia, preoperative biliary decompression, and prolonged duration of surgery were identified as risk factors for POPF. POPF also resulted in increased 90‐day mortality (20%).

**Conclusion:**

POPF remains a potentially life‐threatening complication of pancreaticoduodenectomies. The knowledge and management of modifiable risk factors for this condition may help in mitigating this problem.

## Graphical abstract

Postoperative pancreatic fistula remains a potentially life‐threatening complication of pancreaticoduodenectomies. Preoperative hyperbilirubinemia, pancreatic duct size less than or equal to 3 mm, hypoalbuminemia, preoperative biliary decompression, and prolonged duration of surgery were identified as risk factors for POPF. POPF also resulted in increased 90‐day mortality (20%).The knowledge and management of modifiable risk factors for this condition may help in mitigating this problem.

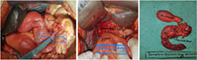

## Introduction

Postoperative pancreatic fistula (POPF) is associated with increased morbidity and postoperative mortality and prolongs intensive care unit (ICU) and hospital stay, which takes its toll on the financial condition of the family and the resources of the hospital.^1^, ^2^, ^3^ A fistula is an abnormal connection between two epithelialized surfaces. A pancreatic fistula can drain a pure pancreatic juice or mixed contents.

POPF is defined as any amount of drain output with amylase content of the drain being three times above the serum amylase levels.^4^ The daily drain effluent determines whether a pancreatic fistula is low or high output. When the drain output is more than 200 mL over 24 h it is termed as high output fistula, and when it is less than 200 mL it is termed as low output fistula.^5^

The 2016 update of the International Study Group (ISGPS) definition of POPFs defines a clinically relevant POPF as drain amount of any output with amylase levels of three times above the serum levels with a clinically relevant condition, which is directly associated with pancreatic fistula. So, grade‐A fistula is considered as biochemical leak as it does not lead to any clinically relevant condition. Grade B requires reposition of drains by endoscopic or percutaneous method or the drains are left in place for more than 3 weeks. Grade C leads to multi‐organ failure and requires surgical intervention.^6^

The known risk factors associated with POPFs are advanced age (age >70 years), soft texture of pancreas, intraoperative blood loss, obstructive jaundice, preoperative biliary stenting, small pancreatic duct diameter, and preoperative malnutrition.^7^, ^8^, ^9^, ^10^, ^11^, ^12^, ^13^

## Methods

This is a single‐institutional, retrospective observational study. We included all consecutive patients who underwent pancreaticoduodenectomy in a specialized surgical unit of a tertiary care referral cancer center in Northeast India. The study period was from 23 April 2012 to 27 December 2019.

### Classification and management

POPF was defined according to the 2016 update of the ISGPS of Pancreatic Fistula. Drain fluid amylase level more than three times the upper limit of the normal value of serum amylase level at the laboratory of our institution was considered as significant. Risk factor variables as given in Table 2 for fistula formation were studied.

The serum and drain fluid amylase levels were analyzed on postoperative days 3, 5, and 7 to confirm or rule out the presence of POPF.

Patients in fistula group‐A (biochemical leak) were managed conservatively, and patients in fistula group‐B were those who needed prolonged drainage of intra‐abdominal amylase‐rich effluents. Surgical exploration was the treatment modality for grade C fistula.

### Pancreaticojejunostomy technique

**Figure 1:**
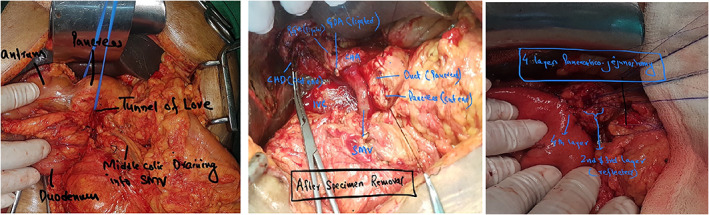
Intraoperative pictures of pancreaticoduodenectomy.

**Figure 2:**
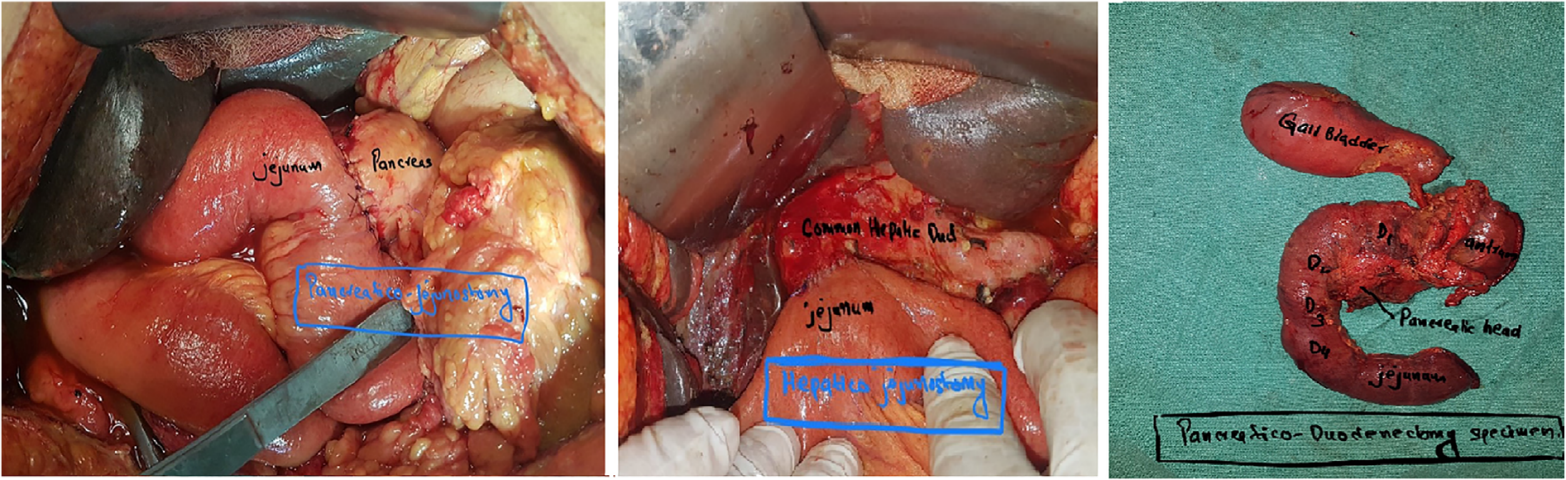
Intraoperative pictures of pancreaticoduodenectomy.

We performed end‐to‐side duct‐to‐mucosa pancreaticojejunostomy anastomosis for all our patients. Duct‐to‐mucosa anastomosis was done with either 4–0 or 5–0 synthetic, absorbable monofilament suture (polydioxanone). Sutures from pancreatic parenchyma and capsule are taken with seromuscular layer of jejunal limb with either polyglactin(vicryl) or polydioxanone suture. The number of sutures depends on size of the duct. An infant feeding tube was used as pancreatic duct stent (Figs 1, 2).

### Statistical analysis

Data were collected from hospital records and computer‐based online hospital reporting system. The statistical analysis was performed using Statistical Package for the Social Sciences (SPSS) statistics version 17.0. Chi‐square tests and Fischer's exact test were applied to categorical variables. *t*‐test was used to calculate mean difference among continuous variables. A *P* value <0.05 was considered statistically significant at 95% confidence interval.

## Results

**Figure 3:**
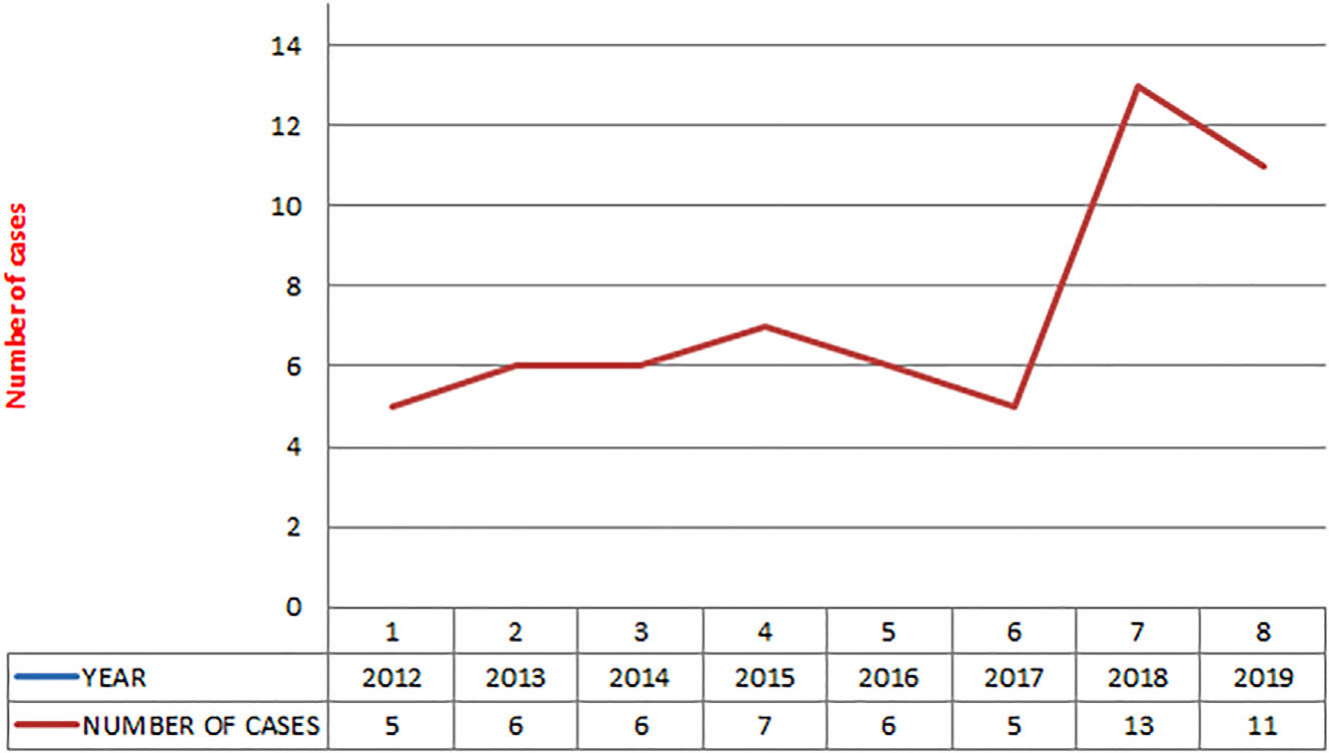
Year‐wise distribution of number of pancreaticoduodenectomies (*n* = 59).

A total of 59 patients underwent pancreaticoduodenectomy during the study period (Fig. 3) with almost equal distribution among males and females (29 and 30 patients respectively). The mean age of the patients was 54.0 years (range 20–72 years). Grade A, B, and C pancreatic fistulas were seen in five (8.5%), three (5.1%), and two (3.4%) patients, respectively (Table 1). Variables for different grades of POPFs in our study population are presented in Table 2.

**Table 1:** Grades of fistula (*n* = 59)

Grade	*n* (%)	Amylase = 3× upper limit of baseline
A (biochemical leak)	5 (8.5%)	Yes
B	3 (5.1%)	Yes
C	2 (3.4%)	Yes

**Table 2:** Variables for different grades of postoperative pancreatic fistulas in study population (*n* = 10)

Grades of pancreatic fistula	Variables
Grade‐A	Amylase levels three times upper limit of normal
Grade‐A	Amylase levels three times upper limit of normal
Grade‐A	Amylase levels three times upper limit of normal
Grade‐A	Amylase levels three times upper limit of normal
Grade‐A	Amylase levels three times upper limit of normal
Grade‐B	Persisting pancreatic drainage >3 weeks, octreotide administration
Grade‐B	Guided drainage of collection, persistent drainage >3 weeks, total parenteral nutrition
Grade‐B	Persistent drainage >3 weeks, octreotide administration
Grade‐C	*Re*‐operation for bleed on postoperative day‐1 (POD‐1), death on POD‐3
Grade‐C	Sepsis with organ failure, postoperative death

Patients in the fistula group had a mean age more than their counterparts who did not develop fistula. The mean age of patients in fistula group was 50.0 years. The mean age of patients without fistula was 46.7 years. Male‐to‐female ratio in the fistula group was 1:1, whereas in without fistula group was 0.9:1.

The mean albumin level in patients in the group of patients who developed fistula was 3.03 g/dL with SD of 0.27, whereas in patients without fistula, it was 3.34 g/dL with SD of 0.44. Albumin level of ≤3.02 ± 0.27 was associated with increased rate of POPF with a *P* value of 0.034, which was statistically significant (Table 3).

**Table 3:** Characteristics of patients (*n =* 59)

Characteristics	Fistula group (*n =* 10)	No fistula (*n =* 49)	*P* value
	*n* (%/SD)	*n* (%/SD)	
Age (mean) in years	50	46.7	
Sex (M:F)	1:1	0.9:1	
Mean albumin (g/dL)	3.02 ± 0.27	3.34 ± 0.4	0.034
Preoperative jaundice	9 (90%)	15 (30.6%)	0.0006
Preoperative biliary decompression	6 (60%)	5 (10.2%)	0.0003
History of transfusion	10%	17.2%	0.5745
Surgery			
Classical	9	48	
Pylorus preserving	1	1	
Pathology			
Periampullary carcinoma	6	35	
Cholangiocarcinoma	2	2	
Carcinoma head of pancreas	2	12	
Duration of surgery (h)	7.3 ± 1.2	5.95 ± 0.9	0.001
Soft pancreatic texture	3 (30%)	5 (10.2%)	0.098
Main pancreatic duct size (mm)			
≤3 mm	6 (75%)	7 (15.6%)	0.0003
>3 mm	2 (25%)	38 (84.4%)	
Blood loss (mL)	805 ± 181.7	451 ± 135.3	0.0001
Postoperative hospital stay (days)	14 ± 3.3	12.7 ± 2.1	0.035

Around 90% of patients with fistula had hyperbilirubinemia compared with 30.6% in those who did not develop POPF. A large number of patients (60% of total) who developed POPF had preoperative biliary decompression in the form of endoscopic retrograde cholangiopancreaticography (ERCP) guided stenting. This is in contrast to only 10.2% of patients in the other group who did not have POPF (Table 3).

The procedure that was performed was invariably classical pancreaticoduodenectomy except in two patients in whom pylorus preserving pancreaticoduodenectomy was done.

Around 30% of patients in the fistula group had soft pancreatic texture compared with 10.2% of patients in the other group. Main pancreatic duct size was ≤3 mm in 75% of patients with fistula group where it was 15.6% in patients with no‐fistula group with a *P* value of 0.0003. The mean duration of surgery was also longer (7.3 ± 1.21 h and 5.95 ± 0.91 h respectively; *P* value of 0.001). It was also noted that the patients in the fistula group had significantly higher intraoperative blood loss (805 ± 181.7 mL and 451 ± 135.3 mL respectively, with a *P* value <0.0001) (Table 3). Preoperative blood transfusion was done in 10% of patients in the fistula group compared with 17.2% of the other group. Patients with fistula had significantly prolonged mean hospital stay (14.7 ± 3.3 days and 12.7 ± 2.1 days respectively, with a *P* value of 0.035 (Table 3).

The coexisting morbidities that were encountered in our patients included delayed gastric emptying, hemorrhage, surgical site infections, and biliary leak. These complications were significantly higher in the group with POPF. The 90‐day mortality in fistula group was 20%, whereas in no fistula group it was 4% (Table 4).

**Table 4:** Complications (*n =* 59)

Complications	Fistula group (*n* = 10)	No fistula group (*n* = 49)	*P* value
	*n* (%)	*n* (%)	
Delayed gastric emptying	8 (80%)	18 (37%)	0.0127
Hemorrhage	3 (30%)	2 (4.1%)	0.0079
Surgical site infections (SSI)	5 (50%)	11 (22.4%)	0.0759
Bile leak	2 (20%)	4 (8.2%)	0.2653
90‐day mortality	2 (20%)	2 (4.1%)	0.071

## Discussion

In our study, the overall incidence of POPF was 17.0% with half of them being biochemical (grade A) leaks not requiring any additional management. As noted previously, the comparison of serum and drain fluid amylase was done on postoperative day 3, day 5, and day 7, following institutional protocol. The mean age of patients in our study for fistula group was 50 years, and that of without fistula was 46.5 years. Thus, the patients with fistula had higher mean age with difference of 3.5 years.

Although not clearly understood, male patients have higher fistula rates.^8^ In our study, however, no sex predilection showed up. In view of low volume of subjects in our study at present, further validation of impact of sex on POPF will be considered with future follow‐up studies from our institute.

Soft pancreatic texture has high risk of POPFs compared with hard pancreas. The studies of Yeo et al. support this as none of their patients out of 53, with hard pancreatic texture developed any fistula, whereas 25% of patients with soft pancreas developed POPFs.^8^ Small‐sized pancreatic duct is also a risk factor for POPFs with ducts less than 3 mm having a higher rate of fistulas.^14^ In our study, pancreatic duct size was available in 53 out of 59 patients, among them 75% of patients in the fistula group had duct size less than or equal to 3 mm, whereas in non‐fistula group, duct size less than 3 mm was seen in 15.6% of patients with a statistically significant *P* value of 0.0003. Soft texture of pancreas posing a higher risk to POPF is also supported in our study, as it was seen in 30% of patients in fistula group, whereas it was only in 10.2% in the non‐fistula group.

There is no consensus regarding the type of anastomotic technique. Retrospective studies, which compared duct‐to‐mucosa anastomosis with dunking technique, did not find any statistically significant POPF rates.^15^ Metanalysis of 2361 patients comparing duct‐to‐mucosa anastomosis with end‐to‐side invagination showed that the latter was associated with increased fistula rates.^9^

Studies of Giacomo Batignani et al. also found similar results with side‐to‐side duct‐to‐mucosa anastomosis having less fistula rate when compared with invagination.^15^ Though previous single institutional studies and meta‐analysis showed that pancreaticogastrostomy was better than pancreaticojejunostomy in terms of leak rate, a recent meta‐analysis established that there were no statistically significant differences between the two techniques for development of POPFs, and mortality and morbidity between two groups were similar.^16^, ^17^, ^18^, ^19^, ^20^ We performed duct‐to‐mucosa pancreaticojejunostomy anastomosis for all our patients uniformly and considering the POPFs noted, we believe that irrespective of the anastomotic technique, multiple factors coexist and play roles in the development of POPF.

Preoperative hyperbilirubinemia is a risk factor for fistula formation. Obstructive jaundice is associated with reduced levels of intestinal bile salts leading to increased gut bacterial microflora and translocation by increased intestinal permeability. Obstructive jaundice is also associated with increased inflammatory response with endotoxemia. Both bacterial translocation and endotoxemia lead to inflammatory cascade and increased infectious complications, which can be a risk factor for POPFs. A total of 90% of our patients in the fistula group had jaundice, whereas that percentage in the non‐fistula group was 30.6%. A study from Hopkins Institute suggested that preoperative biliary drainage was associated with increased biliary fistula rate.^21^ In contrast, Lin et al. indicated that there was no difference in patients with or without preoperative stenting,^22^ and similar findings were reported by Aranha et al.^23^ In our study, preoperative biliary drainage was associated with increased rates of POPF, with 60% of patients in fistula group underwent preoperative decompression, whereas in non‐fistula group only 10.2% underwent preoperative decompression (Table 2). Preoperative biliary drainage along with stenting can cause increased rates of POPFs due to pancreatic ductal inflammation and stent induced duct injury along with contamination of bile and pancreatic fluids.

Our patients underwent classical pancreaticoduodenectomy except in two patients in whom pylorus‐preserving pancreaticoduodenectomy was done. Prolonged surgery and increased blood loss may increase risk for POPFs as these two factors usually indicate the level of complexity of surgery and relative ischemia. With increased mean duration of surgery, there was increased rate of POPF with a significant *P* value of 0.001 in our study.

Five patients with biochemical leaks were conservatively managed and these patients were discharged in healthy condition without any untoward events. In three patients with grade B pancreatic fistula, there was persistent peri‐pancreatic drainage for more than 3 weeks with serum amylase of fluid three times of upper limit of baseline. The drains were in place for more than 3 weeks in all of these patients. They were discharged with the drain once their general condition was acceptable and they were kept under close follow‐up, and the drain was removed at various time period for each patient when the output was negligible.

Twenty percent of patients in the fistula group expired. This included patients who were re‐explored for secondary hemorrhage.

POPF leads to increased morbidity and mortality with prolonged hospital stay, leading to decreased immediate postoperative quality of life apart from increased financial burden to patients and increased hospital resources. In our study, POPF was associated with increased rate of other complications. Postoperative hemorrhage was seen in 30% of patients in the fistula group and 4.1% of patients in the non‐fistula group with a *P* value of 0.0079. Delayed gastric emptying was seen in 80% of patients in fistula group, whereas it was seen in 37% of non‐fistula group with *P* value of 0.0127. The incidence of surgical site infections were also more than double in the fistula group compared with the other group.

Apart from increasing morbidity and immediate postoperative quality of life, POPF also increases mortality rate after pancreaticoduodenectomy. Data from our study also support the above, as 90‐day mortality rate of our patients in fistula group was around 20%, which was five times higher than the other group.

### Surgeon experience and center volume

**Figure 4:**
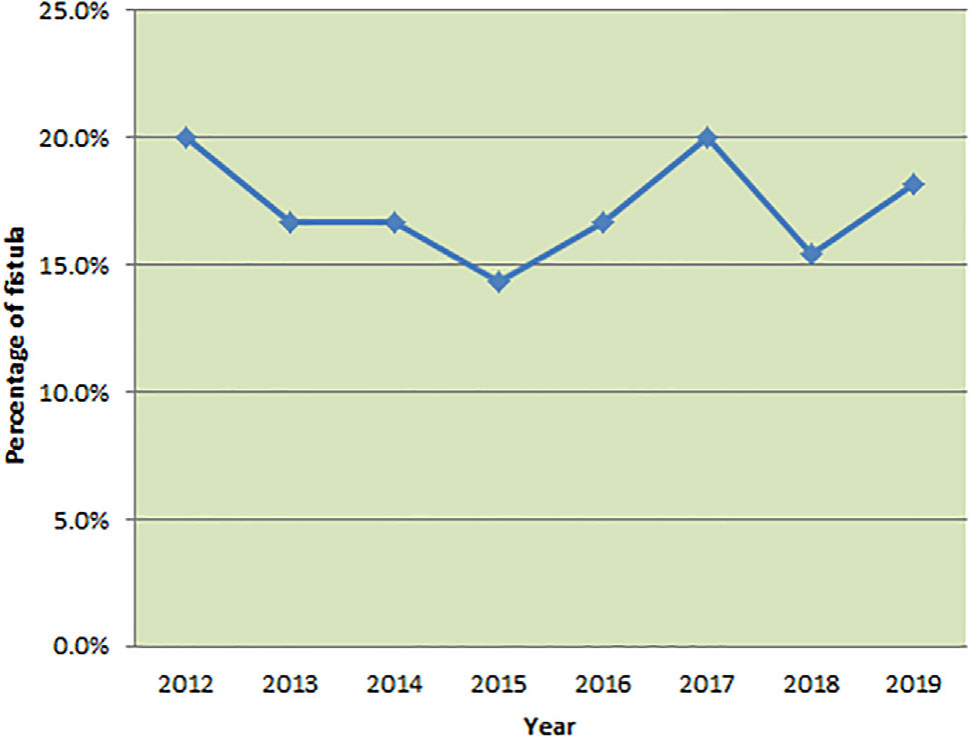
Year‐wise percentage of postoperative pancreatic fistulas.

Our institute is a tertiary cancer care center in Northeast part of India. Majority of hepato‐pancreatico‐biliary cases present in advanced stage at our institute and very low percentage of cases present in operable stages. Most of these surgeries were performed by consultants who were trained at high volume centers for pancreatic surgery. The mean surgical oncology experience of surgeons who performed these surgeries at the end of the study was 17.4 years (range: 8–30 years). The year wise distribution of number of pancreaticoduodenectomies performed and percentage of POPFs are represented in Figures 3 and 4. As seen in Figure 3, the number of cases performed in the last 2 years of study period was doubled from an average of five surgeries per year to 12 cases per year. Residency training program in surgical oncology started at our institute in the year 2016. As seen in Figure 4, there was gradual decrease in fistula rate from 20% in the year 2012 to 14.3% in the year 2015. Though the number of cases increased in year 2018 to 2019, fistula rate again increased to 18.2%. This increased trend could be explained by participation and performance of surgeries by surgical residents who had ≤3 years of experience in surgical oncology and pancreatic surgeries.

The reported postoperative fistula rates at three other centers from India were 15%, 15.39%, and 12% respectively,^24^, ^25^, ^26^ and the fistula rate at our center was 17.0%. Studies have shown that high volume centers and high volume surgeons provide best outcomes after pancreaticoduodenectomy.^25^, ^27^, ^28^ Pancreaticoduodenectomy is associated with major postoperative complications. At high volume centers, apart from availability of enough resources, they are usually equipped with experienced team (critical care team, intervention radiologists, experienced surgeons, and nursing staff), which can improve postoperative outcomes of patients by timely identification and intervention for management of these complications.^29^ A study from India has shown that even at low volume centers, outcomes of patients after pancreatic surgery are minimally affected if hospital is equipped with better infrastructure and experienced team who can manage postoperative complications.^25^ We consider our institute in a transition phase from low volume to high volume center. Though fistula rate at our center was around 17%, about 50% of these cases had only biochemical leak without any change in their postoperative course when compared with no fistula group, and patients with grade‐B fistula were managed successfully with timely intervention in postoperative period.

Our study has helped us in identifying some key factors that can be modified with a goal of achieving favorable perioperative outcomes, especially with respect to POPF rates and severity. Devising and practicing methods in active pursuit of nutritional improvement during the period of evaluation of the patient until the time of surgery in a concerted attempt to optimize the objective indicators like serum albumin level is one of the well understood but very often‐neglected interventions. Although technical difficulties faced during the surgery by virtue of inherent complexities like the tumor and nodal status or the pancreas texture and duct diameter are determinants of duration of surgery, intraoperative blood loss, and outcomes as described above; increased surgical volume is certainly a positive way out of this. Our expectation is to witness decreased rates as our experience grows as a specialized unit.

## Conclusion

POPF remains a potentially life‐threatening complication of pancreaticoduodenectomies. Treatment duration is prolonged with increased financial burden to patients and increased consumption of hospital resources. The knowledge and management of modifiable risk factors for this condition may help in mitigating this problem.

## References

[1] Böttger TC, Junginger T. Factors influencing morbidity and mortality after pancreaticoduodenectomy: critical analysis of 221 resections. World J. Surg. 1999; 23: 164–72. 9880426 10.1007/pl00013170

[2] Cullen J, Surr M, Ilstrup D. Pancreatic anastomotic leak after pancreaticoduodenectomy: incidence, significance and management. Am. J. Surg. 1994; 168: 295–8. 7524375 10.1016/s0002-9610(05)80151-5

[3] van Berge Henegouwen MI, De Wit LT, Van Gulik TM, Obertop H, Gouma DJ. Incidence, risk factors and treatment of pancreatic leakage after a pancreatoduodenectomy: drainage versus resection of pancreatic remnant. J. Am. Coll. Surg. 1997; 185: 18–24. 9208956 10.1016/s1072-7515(97)00007-0

[4] Bassi C, Dervenis C, Butturini G*et al*. Postoperative pancreatic fistula: an international study group (ISGPF) definition. Surgery. 2005; 138: 8–13. 16003309 10.1016/j.surg.2005.05.001

[5] Zinner MJ, Baker RR, Cameron JL. Pancreatic cutaneous fistulas. Surg. Gynecol. Obstet. 1974; 138: 710–2. 4823372

[6] Bassi C, Marchegiani G, Dervenis C*et al*. The 2016 update of the International Study Group (ISGPS) definition and grading of postoperative pancreatic fistula: 11 years after. Surgery. 2017; 161: 584–91. 28040257 10.1016/j.surg.2016.11.014

[7] Suzuki Y, Kuroda Y, Morita A, Pujino Y, Ykawamura T, Saitoh Y. Fibrin glue sealing for the prevention of pancreatic fistula following distal pancreatectomy. Arch. Surg. 1995; 130: 952–5. 7661678 10.1001/archsurg.1995.01430090038015

[8] Yeo CJ, Cameron JL, Lillemoe KD*et al*. Does prophylactic octreotide decrease the rate of pancreatic fistula and other complications after pancreaticoduodenectomy? Results of a prospective randomized placebo controlled trial. Ann. Surg. 2000; 232: 419–29. 10973392 10.1097/00000658-200009000-00014 PMC1421155

[9] Bartoli FG, Arnone GB, Ravera G, Bachi V. Pancreatic fistula and relative mortality in malignant disease after pancreaticoduodenectomy. Review and statistical meta‐analysis regarding 15 years of literature. Anticancer Res. 1991; 11: 1831–48. 1685076

[10] Kanda M, Fujii T, Kodera Y, Nagai S, Takeda S, Nakao A. Nutritional predictors of postoperative outcome in pancreatic cancer. Br. J. Surg. 2011; 98: 268–74. 20960457 10.1002/bjs.7305

[11] Shamali A, De'Ath HD, Jaber B*et al*. Elderly patients have similar short term outcomes and five‐year survival compared to younger patients after pancreaticoduodenectomy. Int. J. Surg. 2017; 45: 138–43. 28782662 10.1016/j.ijsu.2017.07.106

[12] Gaujoux S, Cortes A, Couvelard A*et al*. Fatty pancreas and increased body mass index are risk factors of pancreatic fistula after pancreaticoduodenectomy. Surgery. 2010; 148: 15–23. 20138325 10.1016/j.surg.2009.12.005

[13] Yeh TS, Jan YY, Jeng LB*et al*. Pancreaticojejunal anastomotic leak after pancreaticoduodenectomy multivariate analysis of perioperative risk factors. J. Surg. Res. 1997; 67: 119–25. 9073557 10.1006/jsre.1996.4974

[14] Yang YM, Tian XD, Zhuang Y, Wang WM, Wan YL, Huang YT. Risk factors of pancreatic leakage after pancreaticoduodenectomy. World J. Gastroenterol. 2005; 11: 2456–61. 15832417 10.3748/wjg.v11.i16.2456 PMC4305634

[15] Batignani G, Fratini G, Zuckermann M, Bianchini E, Tonelli F. Comparison of Wirsung‐jejunal duct‐to‐mucosa and dunking technique for pancreatojejunostomy after pancreatoduodenectomy. Hepatobiliary Pancreat. Dis. Int. 2005; 4: 450–5. 16109535

[16] Morris DM, Ford RS. Pancreaticogastrostomy: preferred reconstruction for Whipple resection. J. Surg. Res. 1993; 54: 122–5. 8479169 10.1006/jsre.1993.1018

[17] Yeo CJ, Cameron JL, Maher MM*et al*. A prospective randomized trial of pancreaticogastrostomy versus pancreaticojejunostomy after pancreaticoduodenectomy. Ann. Surg. 1995; 222: 580–8. 7574936 10.1097/00000658-199510000-00014 PMC1234894

[18] Wente MN, Shrikhande SV, Müller MW*et al*. Pancreaticojejunostomy versus pancreatico gastrostomy: systematic review and meta‐analysis. Am. J. Surg. 2007; 193: 171–83. 17236843 10.1016/j.amjsurg.2006.10.010

[19] McKay A, Mackenzie S, Sutherland FR*et al*. Meta‐analysis of pancreaticojejunostomy versus pancreatico gastrostomy after pancreaticoduodenectomy. Br. J. Surg. 2006; 93: 929–36. 16845693 10.1002/bjs.5407

[20] Ramesh H, Thomas PG. Pancreaticojejunostomy versus pancreaticogastrostomy in reconstruction following pancreaticoduodenectomy. Aust. N. Z. J. Surg. 1990; 60: 973–6. 2268215 10.1111/j.1445-2197.1990.tb07516.x

[21] Sohn TA, Yeo CJ, Cameron JL, Pitt HA, Lillemoe KD. Do preoperative biliary stents increase post pancreaticoduodenectomy complications? J. Gastrointest. Surg. 2000; 4: 258–68. 10769088 10.1016/s1091-255x(00)80074-8

[22] Lin JW, Cameron JL, Yeo SJ, Riall TS, Lillemoe KD. Risk factors and outcomes in postpancreatico‐duodenectomy pancreaticocutaneous fistula. J. Gastrointest. Surg. 2004; 8: 951–9. 15585382 10.1016/j.gassur.2004.09.044

[23] Aranha GV, Aaron JM, Shoup M, Pickleman J. Current management of pancreatic fistula after pancreaticoduodenectomy. Surgery. 2006; 140: 561–8. 17011903 10.1016/j.surg.2006.07.009

[24] Shah OJ, Singh M, Latto MR, Bangri SA. Pancreaticoduodenectomy: a study from India on the impact of evolution from a low to a high volume unit. World J. Gastrointest. Surg. 2016; 8: 583–9. 27648163 10.4240/wjgs.v8.i8.583 PMC5003938

[25] Vinchurkar K, Pattanshetti VM, Togale M, Hazare S, Gokak V. Outcome of pancreaticoduodenectomy at low volume centre in tier II city of India. Indian J. Surg. Oncol. 2018; 9: 220–4. 29887705 10.1007/s13193-018-0744-8 PMC5984862

[26] Shukla PJ, Barreto SG, Bedi MMS*et al*. Perioperative outcomes of pancreatoduodenectomy in India: a multicenter study. HPB. 2009; 11: 638–44. 20495631 10.1111/j.1477-2574.2009.00105.x PMC2799616

[27] Toomey PG, Teta AF, Patel KD, Ross SB, Rosemurgy AS. High‐volume surgeons vs high‐volume hospitals: are best outcomes more due to who or where? Am. J. Surg. 2016; 211: 59–63. 26542187 10.1016/j.amjsurg.2015.08.021

[28] Hata T, Motoi F, Ishida M*et al*. Effect of hospital volume on surgical outcomes after pancreaticoduodenectomy: a systematic review and meta‐analysis. Ann. Surg. 2016; 263: 664–72. 26636243 10.1097/SLA.0000000000001437

[29] Merrill AL, Jha AK, Dimick JB. Clinical effect of surgical volume. N. Engl. J. Med. 2016; 374: 1380–2. 27050211 10.1056/NEJMclde1513948

